# Urine BLCA-4 exerts potential role in detecting patients with bladder cancers: a pooled analysis of individual studies

**DOI:** 10.18632/oncotarget.6061

**Published:** 2015-10-09

**Authors:** Qiliang Cai, Yudong Wu, Zhanjun Guo, Rui Gong, Yang Tang, Kuo Yang, Xiaodong Li, Xuemei Guo, Yuanjie Niu, Yan Zhao

**Affiliations:** ^1^ Department of Urology, Tianjin Institute of Urology, the Second Hospital of Tianjin Medical University, Tianjin, China; ^2^ Pharmaceutical Department, the Second Hospital of Tianjin Medical University, Tianjin, China; ^3^ Department of Radiotherapy, the Second Hospital of Tianjin Medical University, Tianjin, China; ^4^ Library of Tianjin Medical University, Tianjin Medical University, Tianjin, China; ^5^ Tianjin Institute of Infectious Disease, the Second Hospital of Tianjin Medical University, Tianjin, China

**Keywords:** BLCA-4, diagnostic biomarker, bladder cancer, diagnosis, meta-analysis

## Abstract

Epidemiological studies have explored the diagnostic effect of urine BLCA-4 in bladder cancer. However, the results remain controversial. Therefore, we conducted this pooled analyses to determine the overall accuracy of urine BLCA-4 in bladder cancer. A comprehensive electronic and hand search was conducted for related literatures though several databases. QUADAS-2 was used to assess the quality of each included studies. Diagnostic parameters were calculated using Meta-Disc (version 1.4) and Stata (version 12.0) software. Nine published articles with 1,119 subjects were included. The summary estimates were: sensitivity 0.93 (95% confidence interval [CI] = 0.90-0.95), specificity 0.97 (95% CI, 0.95-0.98), positive likelihood ratio 48.16 (95% CI, 11.77-197.01), negative likelihood ratio 0.08 (95% CI, 0.06-0.11), diagnostic odds ratio 534.03 (95% CI, 150.15-1899.31), and the AUC was 0.9607. In conclusion, urine BLCA-4 is a promising marker in diagnosing bladder cancer.

## INTRODUCTION

Cancer is a major public health problem and the second leading cause of death in the United States, and is supposed to surpass heart diseases to be the leading cause of death in the next few years [1]. Bladder cancer is the fourth most common cancer and the eighth most common cause of cancer-specific mortality in United States men. In 2015, approximately 56,230 new cases of bladder cancer with incidence rate of 7%, and 11,510 deaths with incidence rate of 4%, resulted from this disease among United States men [1]. As of now, cigarette smoking, chemical materials, chronic inflammation or infection of the bladder, spinal cord injuries, age, and diet were considered as the risk factors [2, 3, 4]. Simultaneously, 90% was histologically confirmed as transitional cell carcinomas, about 5% was squamous cancers, and 1% was adenocarcinomas [5]. Non-muscle invasive bladder cancer accounts for 75%-85% (70% are Ta, 20% are T1, and 10% are carcinoma *in situ* lesions), and the recurrence rate for Ta patients from 50% to 70%, while 10-30% T1 or carcinoma *in situ* patients progress to muscle invasion over a 5-yr period [6, 7, 8]. When bladder cancer is detected early at a localized stage, the 5-year survival rate is 94%. Disease that has spread regionally or distantly lowers survival to 49 and 6%, respectively [9]. Although hematuria can be found in approximately 80% bladder cancer patients, it is not considered as the special symptom of the disease. Urine hematuria can be found in cystitis, infection of unary system organs, urinary stones, and patients after taking some special drugs. Moreover, other symptoms, such as painful or difficult urination, increased frequency of urination or abdominal pain were neither considered to be the diagnostic standard for bladder cancer [10]. Considering the lack of symptoms, high recurrent ratio, bad prognosis of the disease, it is crucial to find a high sensitive and specific detection tool at the early stage.

Currently, cystoscopy, cytology and imaging of the upper urinary tract are the main methods used to detect bladder cancer patients. Cystoscopy, as the gold standard for decades, can identify most papillary and solid lesions, wildly be used in bladder cancer recurrence detection [11], but it can miss certain lesions, in particular small areas of carcinoma *in situ* (CIS). Furthermore, it is an invasive procedure and it can result expensive for most patients affected by bladder cancer, which widely limit its use for both early detection and long-life follow up of bladder cancer. An ideal method to detect bladder cancer should be not only relatively inexpensive and noninvasive, but also have a good sensitivity and high specificity; not only can be used in low-grade but also high-grade bladder cancer. Cytology, considered to be the second gold standard, a urine-based noninvasive test, has a median specificity of 94%, but a median sensitivity of less than 44% [12], ranging only from 4% to 31% [13], particularly for low-grade bladder cancers [14]. Thus, a noninvasive, highly sensitive, and speciﬁc urine-based marker, better than urine cytology, for detecting bladder cancer should be explored.

BLCA-4, as one of the six nuclear matrix proteins (NMPs), was firstly identified in 1996[15]. It can be found in the early stage of bladder cancer, but not expressed in normal tissues [16, 17], which was reported to be the most sensitive and speciﬁc urinary marker [15, 18] with a sensitivity range from 89%-96.4% and specificity range from 95%-100% [17, 19-23]. Preliminary studies have explored the diagnostic performance of BLCA-4 in patients with bladder cancer, but with elusive results. Therefore, we search all eligible studies to summarize the diagnostic value of BLCA-4 for bladder cancer.

## RESULTS

### Literature search outcome

**Figure 1:**
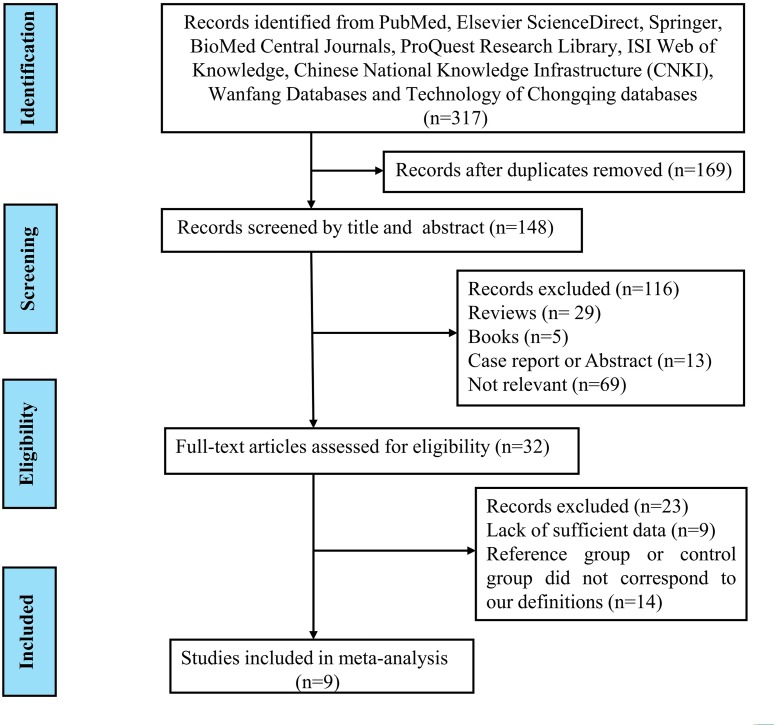
Flow chart describing the systematic literature search and study selection process

A comprehensive computer literature search was performed by independent reviewers from PubMed, Elsevier ScienceDirect, Springer, BioMed Central Journals, ProQuest Research Library, ISI Web of Knowledge, Chinese National Knowledge Infrastructure (CNKI), Wanfang Databases and Technology of Chongqing databases. Firstly, 317 literatures were retrieved. 169 records were excluded after discarding duplicates and 148 studies remained and were screened on title and abstract for eligibility in this meta-analysis. 33 studies were potentially relevant after reviewing the titles and abstracts, which were then evaluated in detail. After reviewing the full article, 22 studies were excluded for 13 of them did not correspond to our definition of reference and control group, while the other studies were lack of sufficient data to fulfill the 2×2 table. Finally, nine studies fulfilled our inclusion criteria and were included in our meta-analysis [19, 20, 23, 29-34]. A ﬂowchart describing the process of selecting studies is shown in Figure 1.

### Study characteristics and quality assessment

**Figure 2:**
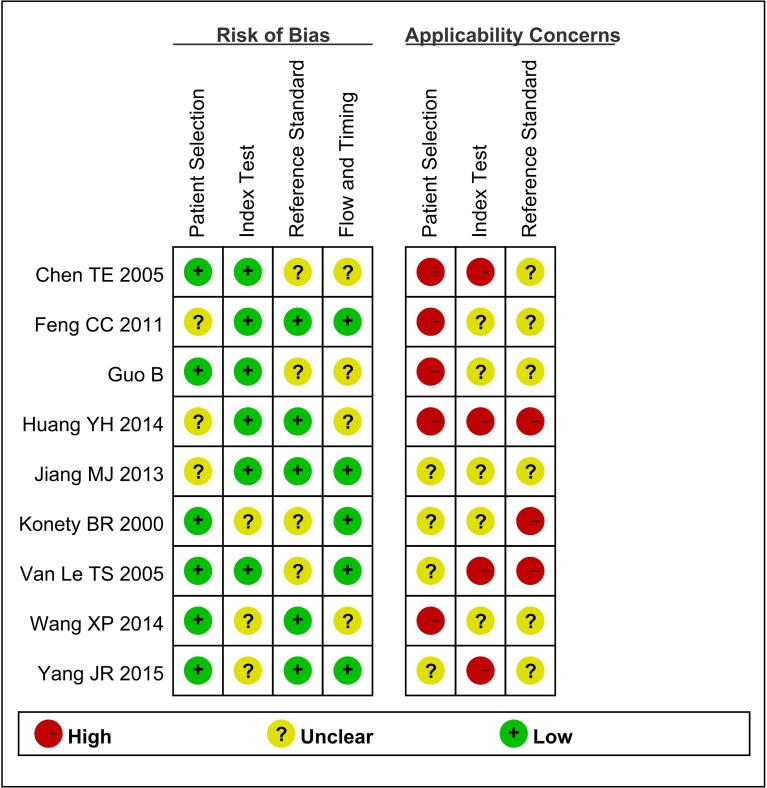
Summary the assessment of methodological quality of included studies by QUADAS-2 tool

Baseline characteristics of the eligible studies are summarized in Table 1. A total of nine studies with 1,119 subjects were included in our meta-analysis study. Among these studies, two of nine were performed in USA [19, 20], and the rest seven were conducted in China [23, 29-34]. All eligible studies were published between 2000 and 2015. The sample size ranges from 76 to 180. Urine BLCA-4 was detected by various kinds of methods. ELISA test was applied in seven studies [20, 23, 30-34], and the other two studies used Sandwich immunoassay [19] and QPCR [29], respectively. Additionally, vary cut off values were applied, the one used 0.04 (OD) [19], the other one used 1.7×10^−4^ (A) [23], while the rest studies used 13 (A/ug) [20, 29-34]. Notably, key data was successfully extracted from all included studies, such as, true positive (TP), false positive (FP), false negative (FN), and true negative (TN). The number of TP ranged from 28 to 75 while the number of FN ranged from 0 to 8. The number of FP ranged from 0 to 14 while the number of TN ranged from 25 to 110. QUADAS-2 summary plot was presented in Figure 2. As shown, methodological quality of eligible studies was adequate and not significantly affected by bias.

**Table 1:** Characteristics of the nine include studies in this meta-analysis

Study	Country	Year	Sample size	Sample type	Assay method	Cut off value	TP	FP	FN	TN
Konety BR	USA	2000	106	Urine	ELISA test	13 A /ug	53	0	2	51
Van Le TS	USA	2005	140	Urine	Sandwich immunoassay	OD=0.04	67	3	8	62
Chen TE	China	2005	76	Urine	ELISA test	13 A /ug	33	0	2	41
Guo B	China	2011	155	Urine	QPCR	13 A /ug	65	14	7	69
Feng CC	China	2011	136	Urine	ELISA test	1.7×10^−4^A	74	0	2	60
Jiang MJ	China	2013	88	Urine	ELISA test	13 A /ug	28	0	2	58
Huang YH	China	2014	82	Urine	ELISA test	13 A /ug	49	1	7	25
Wang XP	China	2014	156	Urine	ELISA test	13 A /ug	42	0	4	110
Yang JR	China	2015	180	Urine	ELISA test	13 A /ug	75	0	5	100

ELISA, Enzyme Linked Immunosorbent Assay; QPCR, Quantitative Polymerase Chain Reaction; OD, Absorbance units; TP, true positive; FP, false-positive; TN, true negative; FN, false-negative.

### Diagnostic accuracy and threshold analysis

**Figure 3:**
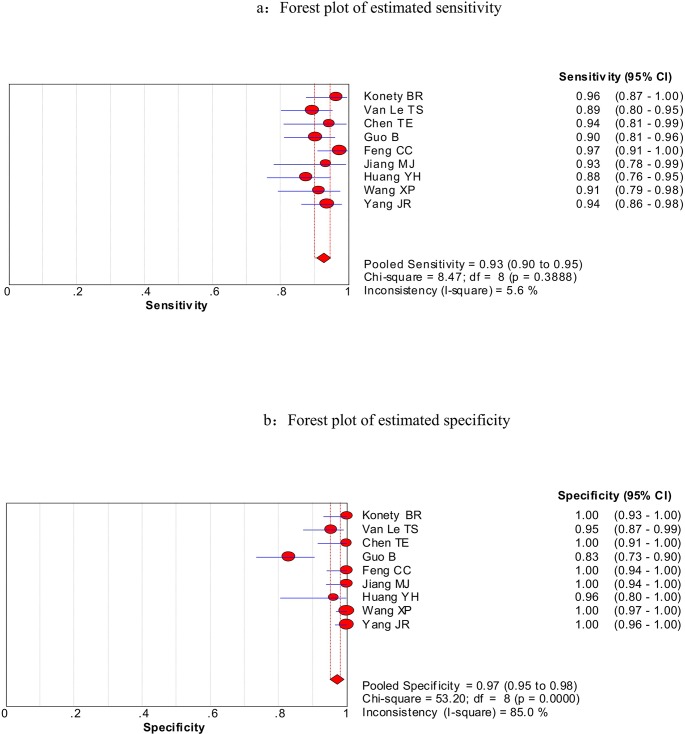
Forest plots of estimated sensitivity (a) and specificity (b) for urine BLCA-4 in the diagnosis of bladder cancer

**Figure 4:**
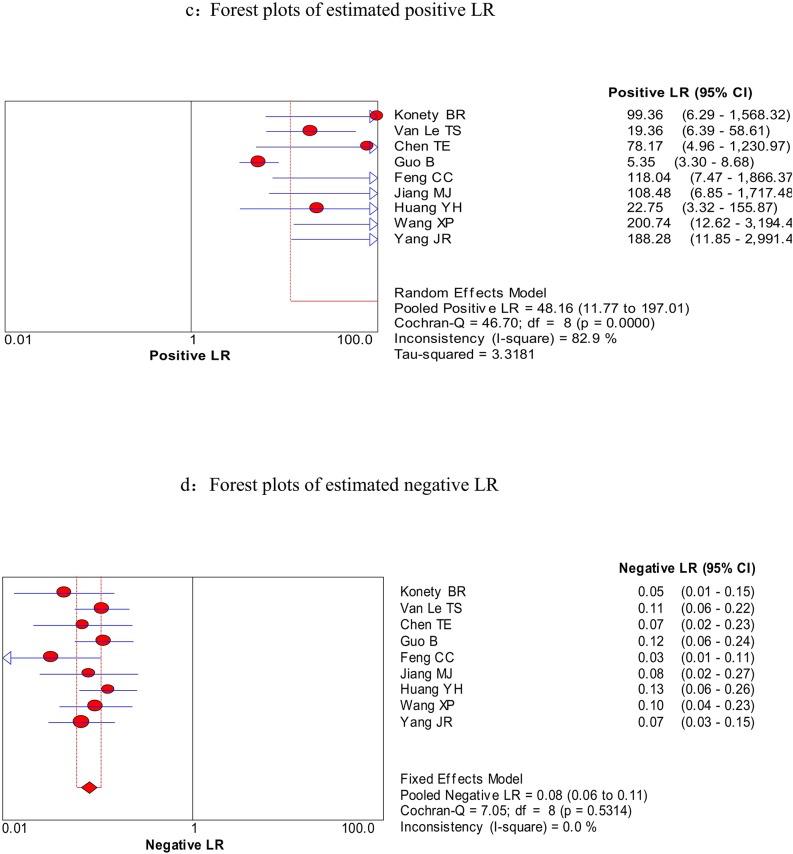
Forest plots of estimated PLR (a) and NLR (b) for urine BLCA-4 in the diagnosis of bladder cancer

**Figure 5:**
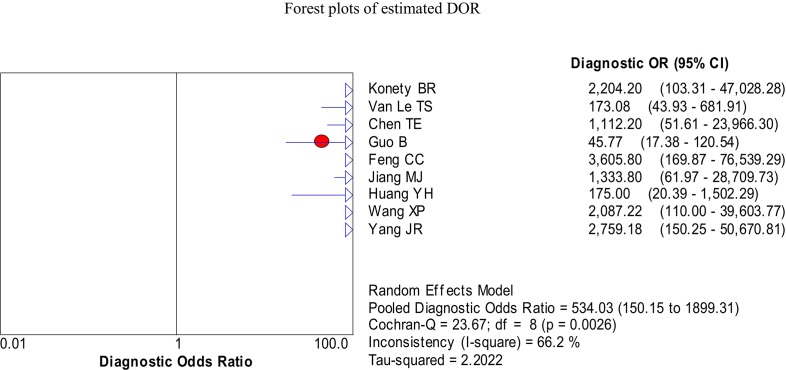
Forest plots of the pooled diagnostic odds ratio (DOR) for urine BLCA-4 in the diagnosis of bladder cancer of the included nine studies

**Figure 6:**
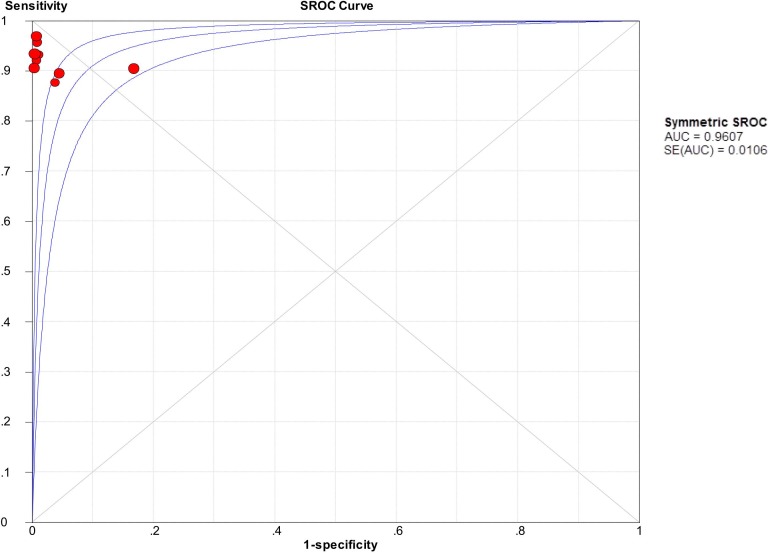
Summary receiver operating characteristic (SROC) curve for urine BLCA-4 in the diagnosis of bladder cancer of the included nine studies

We firstly used spearman approach to explore whether the threshold effect was existed in our work, because it is the important source of heterogeneity. The Spearman correlation coefficient of sensitivity and 1-specificity in this meta-analysis was −0.567 with a p- value of 0.112, suggesting that there is no heterogeneity from threshold effect. The heterogeneity was measured by Q test and the inconsistency index (*I^2^*) to choose the appropriate calculation model. There were statistically significant heterogeneity in pooled specificity (*I^2^* = 85%, *P* = 0.000), pooled positive likelihood ratio (PLR) (*I^2^* = 82.9%, *P* = 0.000) and pooled DOR (*I^2^* = 66.2%, *P* < 0.003), respectively. Therefore, the random effects model was used for calculating specificity, PLR and DOR. Based on the extracted data of TP, TN, FP, and FN from the included studies evaluated the diagnostic accuracy of BLCA-4 in bladder cancer, we get the following diagnostic quantitative results. The pooled sensitivity and specificity were 0.93 (95% CI, 0.90-0.95, Figure 3a) and 0.97 (95% CI, 0.95-0.98, Figure 3b), respectively. The pooled PLR and NLR were 48.16 (95% CI, 11.77-197.01, Figure 4a) and 0.08 (95% CI, 0.06-0.11, Figure 4b), respectively. The pooled DOR was 534.03 (95% CI, 150.15-1899.31, Figure 5), and the SROC curve for BLCA-4 is positioned near the desirable upper left corner; while the area under the curve (AUC) was 0.9607, indicating that the level of overall accuracy was high (Figure 6). Furthermore, subgroup analysis was performed by ethnicity, sample size, assay method and cut off value, and the results was summarized in Table 3.

### Meta-regression analysis and publication bias

Heterogeneity was found in the summary estimates of sensitivity, specificity, PLR, NLR, and DOR. Thus, meta-regression was conducted to explore the source of heterogeneity based on country, sample size, cut off value and assay method. However, none of the above covariates contributed heterogeneity (all *P* > 0.05) (Table 2). Although subgroup analysis was performed, no parameters presented the source of heterogeneity. Deeks' funnel plots was explored to detect the possible publication bias, and no significant publication bias was found in the meta-analysis (*P* = 0.972) (Figure 4).

**Table 2:** Results of the multivariable meta-regression model for the characteristics with backward regression analysis (Inverse variance weights; variables were retained in the regression model if P<0.05)

Variables	Coeff.	Std.Err	*P*-value	RDOR	[95%CI]
Cte.	4.459	2.0300	0.1155	---	---
S	−0.094	1.0936	0.9372	---	---
Country	−0.024	1.2802	0.9865	0.98	(0.02;57.43)
Sample size	0.019	0.0205	0.4246	1.02	(0.95;1.09)
Cut off value	0.015	0.1013	0.8950	1.01	(0.74;1.40)
Assay method	−1.860	1.7688	0.3702	0.16	(0.00;43.34)

Cte: Constant Coefficient; S: Statistic S; RDOR: Relative diagnostic odds ratio.

**Table 3:** Summary results of diagnostic accuracy of urine BLCA-4 for bladder cancer

Subgroup	No. of Studies (No. of cases)	Sensitivity (95% CI)	Specificity (95% CI)	PLR (95% CI)	NLR (95% CI)	DOR (95% CI)	AUC
Ethnicity							
USA	2 (246)	0.92 (0.86, 0.96)	0.97 (0.93, 0.99)	30.47 (10.90, 85.19)	0.08 (0.05, 0.15)	301.54 (91.12, 997.85)	--
China	7 (873)	0.93 (0.90, 0.95)	0.97 (0.95, 0.98)	56.61 (7.60, 421.82)	0.08 (0.06, 0.11)	631.46 (117.35, 3397.91)	0.9622
Sample size							
> 100	6 (873)	0.93 (0.90, 0.95)	0.96 (0.94, 0.98)	47.67 (7.55, 301.02)	0.08 (0.06, 0.11)	583.35 (105.68, 3219.94)	0.9623
≤ 100	3 (246)	0.91 (0.84, 0.95)	0.99 (0.96, 1.00)	48.12 (13.15,176.07)	0.09 (0.05, 0.17)	444.90 (97.10, 2038.54)	0.8594
Assay method							
Sandwich immunoassay	1 (140)	--	--	--	--	--	--
QPCR	1 (155)	--	--	--	--	--	--
ELISA test	7 (824)	0.94 (0.91, 0.96)	1.00 (0.99, 1.00)	91.56 (35.79, 234.23)	0.07 (0.05, 0.10)	1094.47 (367.11, 3262.95)	0.9780
Cut off value							
0.04 (OD)	1 (140)	--	--	--	--	--	
1.7×10^−4^ A	1 (136)	--	--	--	--	--	
13 A /ug	7 (843)	0.92 (0.89, 0.95)	0.97 (0.95, 0.98)	55.03 (7.66, 395.23)	0.09 (0.07, 0.13)	583.04 (113.54, 2993.93)	0.9612
Total	9(1119)	0.93 (0.90, 0.95)	0.97 (0.95, 0.98)	48.16 (11.77, 197.01)	0.08 (0.06, 0.11)	534.03 (150.15, 1899.31)	0.9607

ELISA, Enzyme Linked Immunosorbent Assay; QPCR, Quantitative Polymerase Chain Reaction; OD, Absorbance units; CI, confidence interval; PLR, positive likelihood ratio; NLR, negative likelihood ratio; DOR, diagnostic odds ratio; AUC, area under the curve.

## DISCUSSION

Up to date, cystoscopy has always been considered the gold method to detect bladder cancer, and also to be used to follow up patients who underwent tumor excision surgery. However, it is an invasive tool and very expensive for some patients. In addition, although urine cytology examination was widely used in clinical deeds, it is of low sensitivity, ranging from 4% to 31% [13], which limits its use. As such, finding a urine-based noninvasive, inexpensive, rapid, accurate and easy to administer and interpret, with high sensitivity and specificity is a hot topic among urologists and related researchers.

BLCA-4 is a nuclear transcription factor expressed in bladder tumors, especially in very early stage of tumorigenesis of the disease. That is, it has a high sensitivity of detecting low-grade bladder cancer, but not found in normal bladder tissues. Preliminary studies have explored the diagnostic accuracy of urine BLCA-4 in bladder cancer, but the results are inconclusive. Therefore, we performed this pooled analyses to determine the diagnostic role of urine BLCA-4. In the present study, we performed a comprehensive databases search for all the eligible studies reported the diagnostic function of urine BLCA-4 for bladder cancer. After pooling all the data, we get a summary of diagnostic parameters as follows. The pooled sensitivity was 0.93 (95% CI, 0.90-0.95) and the pooled specificity was 0.97 (95% CI, 0.95-0.98), which represent a promising diagnostic marker in bladder cancer. To the best of our knowledge, summary PLR > 10 has great power to approve the diagnosis of a disease, while summary NLR < 0.1can negate a diagnosis of a disease. In our study, the pooled PLR was 48.16, and the pooled NLR was 0.08, indicating that the urine BLCA-4 test exerts important function in diagnosing bladder cancer.

DOR represents the “discrimination” ability of diagnostic test. It ranges from 0 to infinity, and also can be considered as the greater of the DOR is, the stronger of the discriminative ability is. Based on the theory mentioned above and the pooled DOR value is 534.03 of our work, it demonstrated that the urine BLCA-4 could be a useful biomarker in diagnosing bladder cancer. SROC is used to summarize overall test performance, and AUC is another parameter to evaluate the diagnostic value. Statistically, if the AUC in the region of 0.97 or above is considered to have excellent accuracy; an AUC of 0.93 to 0.96 is very good, an AUC of 0.75 to 0.92 is good, and an AUC of less than 0.75 should be cautiously evaluated for the test may have obvious deficiencies in accuracy and is approaching the random test [30, 31]. Our data showed that urine BLCA-4 has good accuracy in diagnosing bladder cancer with an AUC of 0.9607.

Substantial heterogeneity was found in our present meta-analysis during analyzing the pooled specificity, PLR, NLR, and DOR. Therefore, random-effect model was used to synthesize the data. To our knowledge, heterogeneity is an important factor that could interpret the result of the meta-analysis. So, we explored Spearman approach to clarify if the threshold effect contributed to the source of heterogeneity, and the Spearman correlation coefﬁcient was −0.567, p value was 0.112, which demonstrated that the heterogeneity among included studies could not be induced by the threshold effect. For instance, we further adopted meta-regression to explore the source of heterogeneity, based on country, cut off value, sample size and assay method parameters, and the results indicated that no parameters mentioned above could explain that (all *P* value > 0.05).

Some possible limitations existed in this meta-analysis should be acknowledged. On the one hand, relative small numbers of included studies, and less than 100 subjects were researched in three of 11 studies. As we all known, meta-analysis has great power to get a relative precise result thorough pooling all related data, the larger of sample is, the more precise result we will get. On the other hand, heterogeneity was found in this work. Although Spearman approach was performed to verify whether the threshold effect was the source of heterogeneity, and then, meta-regression was conducted. Unfortunately, no parameters analyzed above were found to be the source of heterogeneity. What's more, only literatures in English and Chinese were included in this present study. Further studies with different ethnic subjects are needed to verify our results.

In summary, our current study suggests that BLCA-4 has good diagnostic accuracy for bladder cancer. Nevertheless, more well-designed prospective, large-scale and multicenter validation clinical studies are also needed to evaluate the diagnostic role of BLCA-4 in patients with bladder cancer.

**Figure 7:**
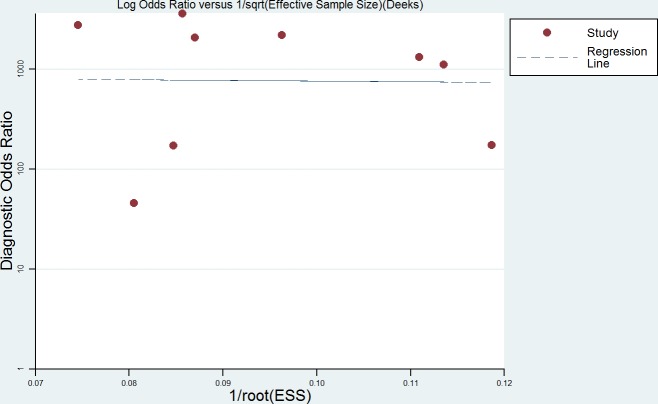
Deek's Funnel Plot Asymmetry Test for the assessment of potential publication bias

## MATERIALS AND METHODS

Our present meta-analysis was performed in accordance with the Preferred Reporting Items for Systematic reviews and Meta-Analyses (PRISMA) guidelines [24].

### Literature search strategy

PubMed, Elsevier ScienceDirect, Springer, BioMed Central Journals, ProQuest Research Library, ISI Web of Knowledge, Chinese National Knowledge Infrastructure (CNKI), Wanfang Databases and Technology of Chongqing databases were searched by two independent researchers to identify relevant studies that evaluated the diagnostic value of BLCA-4 for bladder cancer patients, up to June 26, 2015. The following search terms (in Title, Abstract or keywords fields) were used as follows: “bladder cancer or bladder carcinoma or bladder tumor”, “Bladder cancer specific nuclear matrix proteins or BLCAs or BLCA-4”, “diagnosis”, “sensitivity” and “specificity”. Additionally, we also conducted a manual search by two researchers for other relevant studies from the reference lists of all identified articles. And then, all identified titles, abstracts and manuscripts were independently reviewed by two researchers mentioned above to determine if a study was suitable for the present meta-analysis. In order to minimize potential publication bias, we didn't set restriction on time period, sample size, population, language, or type of report.

### Literature selection criteria

Studies included in present quantitative analyses should meet the following criteria: (1) case-control or cohort design; (2) diagnostic effect about BLCA-4 for bladder cancer; (3) bladder cancer was diagnosed based on histological examination; (4) ELISA or immunohistochemical was adopted as a reference standard; (5) The levels of BLCA-4 in urine was determined; (6) Sensitivity, speciﬁcity, and cut-off values can be found in identified studies or calculated from the provided data. While the exclusion criteria were listed as follows: (1) Not case-control or cohort design studies; (2) studies not related to the human; (3) studies with insufficient data to construct the 2×2 table; (4) The reference standard was not ELISA or immunohistochemical; (5) Meta-analyses, reviews, letters, comments, editorial articles and conference abstracts; and (6) publications were identified as duplicates. All records were reviewed by two authors independently and reached consensus at each eligible study. If studies had overlapping subjects, only the study with the largest sample size was included in the ﬁnal analysis.

### Data extraction and quality assessment

Relevant data was extracted by two reviewers independently from the full text of each identified study using a standardized form. Another reviewer will rejoined if there are disagreements existed between the two reviewers, and the majority opinion was used to resolve disagreements between them. To perform validity analyses, the following information was obtained from each identified article: author's name, journal and year of publication, country of origin, detection method, number of samples, and number of samples with the indicated results (including true positive [TP], true negative [TN], false negative [FN] and false positive [FP]). The cut-off levels of BLCA-4 were also extracted from the articles. If several cut-offs were adopted in one study, the best test performance one will be chosen. If several detection methods were used in one study, we chose the results that were obtained with the most sensitive method. Quality assessment of diagnostic accuracy studies 2 (QUADAS-2), which is a tool for the quality assessment of studies of diagnostic accuracy, was explored to evaluate the quality of the eligible studies included in this meta-analysis [25].

### Statistical analysis

The meta-analyses were performed according to the standard methods recommended for the diagnostic accuracy of meta-analyses was used [26]. The following parameters representing test accuracy were calculated based on the data (TP, FP, FN, and TN) we extracted from each included studies: the pooled sensitivity, specificity, positive likelihood ratio (PLR), negative likelihood ratio (NLR), diagnostic odds ratio (DOR), and corresponding 95% confidence intervals (95% CI). The summary receiver operative curve (SROC), which shows the relationship between sensitivity and 1-speciﬁcity, was used to evaluate the consistency of results among all studies as well as the accuracy of the test. Simultaneously, the area under the SROC curve (AUC) was also calculated. The heterogeneity was measured by Q test and the inconsistency index (*I^2^*), and a *P* < 0.05 and a *I^2^* > 50% indicated significant heterogeneity among studies, the random-effect model (DerSimonian-Laird method) was conducted for the meta-analysis to calculate the pooled sensitivity, specificity, and other related indexes of the studies, and meta-regression was performed to detect the source; otherwise, the fixed-effect model (Mantel-Haenszel method) was chosen.

In addition, the Spearman correlation coefficient was used to verify if the heterogeneity in meta-analysis could be explained by a threshold effect; a threshold effect was defined as a positive correlation (*P* < 0.05). Sub-group analyses were performed for sample size, countries, detection methods, and TNM stages. Deek's Funnel Plot Asymmetry Test [27] was applied to determine the presence of publication bias using STATA 12.1 software (Stata Corp., College Station, Texas, USA.) [27], and a *P* < 0.05 indicated the presence of publication bias. Meta-Disc (version 1.4) software [28] was also used to calculate the other parameters of diagnostic accuracy. All P values were two-sided, and *P* < 0.05 were considered statistically signiﬁcant.
